# The usefulness of combined analysis using CIScore and VSRAD parameters for differentiating between dementia with Lewy body and Alzheimer’s disease

**DOI:** 10.1007/s11604-024-01604-5

**Published:** 2024-06-10

**Authors:** Gaku Honda, Shigeki Nagamachi, Mai Takahashi, Yukie Higuma, Tomonobu Tani, Kosuke Hida, Kengo Yoshimitsu, Koji Ogomori, Yoshio Tsuboi

**Affiliations:** 1 Department of Radiology, Faculty of Medicine, Fukuoka University, Fukuoka, Japan; 2 Department of Psychiatry, Faculty of Medicine, Fukuoka University, Fukuoka, Japan; 3 Department of Neurology, Faculty of Medicine, Fukuoka University, Fukuoka, Japan

**Keywords:** Dementia with Lewy bodies, Alzheimer’s disease, eZIS, VSRAD

## Abstract

**Purpose:**

The Cingulate Island score (CIScore) is useful index for differentiating between dementia with Lewy body (DLB) and Alzheimer’s disease (AD) using regional cerebral blood flow (rCBF) SPECT. The Z score standing for medial temporal lobe (MTL) atrophy and the ratio of Z score between dorsal brain stem (DBS) to MTL are useful indices for differentiating between DLB and AD using MRI with VSRAD. The current study investigated the diagnostic ability by the combined use of rCBF SPECT and MRI in the differentiation between AD and DLB.

**Materials and methods:**

In cases with 42 AD and 28 DLB undertaken Tc-99m-ECD SPECT and MRI, we analyzed differential diagnostic ability between AD and DLB among following conditions by single or combined settings. Namely, they were (1) the CIScore as a parameter of rCBF SPECT (DLB ≦ 0.25), (2) Z score value of MTL atrophy (DLB ≦ 2.05), (3) the ratio of Z score of DBS to medial temporal gray matter as a parameter of brain atrophy using VSRAD (DLB ≧ 0.38). Also, we analyzed them both including and omitting the elderly (over 75 years old).

**Results:**

The accuracy of differential diagnosis in this condition was 74% for (1), 69% for (2), and 67% for (3). The accuracy by combination condition was 84% for (1) and (2), 81% for (1) and (3), and 67% for (2) and (3), respectively. The combination method by CIScore and the Z score of MTL showed the best accuracy. When we confined condition to ages younger than 75 years, the accuracy improved to 94% in the combination method.

**Conclusion:**

The combined use of CIScore and Z score of MTL was suggested to be useful in the differential diagnosis between DLB and AD particularly in younger than 75 years old.

**Supplementary Information:**

The online version contains supplementary material available at 10.1007/s11604-024-01604-5.

## Background

Both Alzheimer’s disease (AD) and dementia with Lewy bodies (DLBs) are main two dementia with high incidence rate and their increase is social problem in the worldwide [1]. Because many commonly used medications produce severe side effects in patients with DLB, such as medications with anticholinergic or antidopaminergic actions, without the correct differential diagnosis [2].

Although both amyloid positron emission tomography (PET) and Tau PET have emerged as powerful diagnostic tools [3–5], these modalities are only available in larger, specialized hospitals and research centers. In addition, there are rigorous of insurance coverage for amyloid PET, and it is not performed in still in all suspected AD cases. On the other hand, FDG-PET serves as a valuable tool for measuring brain glucose metabolism, enabling the differentiation of conditions by assessing hypometabolism in the occipital lobe in DLB [6, 7]. Additionally, regional cerebral blood flow (rCBF) SPECT offers a more convenient and practical imaging method suitable for actual clinical settings [8, 9].

The volumetric MRI is extensively used to diagnose AD using the patterns of cerebral atrophy, involving the medial temporal lobe (MTL) and temporoparietal association cortices [10]. In Japan, MRI is used more often than SPECT in many hospitals.

In the differential diagnosis between DLB and AD rCBF SPECT analysis of both posterior-cingulate gyri and occipital lobes is useful, which is known as the Cingulate Island Sign score (CIScore) [11–13]. Although the sign is originally reported by FDG-PET [14], it is also widely used in routine clinical practice by rCBF SPECT examination recently, and the usefulness is confirmed [11]. The diagnostic ability of CIScore is known to be higher in conditions without influence of brain atrophy by aging [13].

In MRI, the Z score standing for MTL atrophy and the ratio of Z score between dorsal brain stem (DBS) CBF to MTL are useful indices for differentiating between DLB and AD using VSRAD [10]. The atrophic change in MTL is prominently noted in AD compared to DLB, and DBS is significantly noted in DLB compared to AD [10].

Thus, there are many reports regarding the diagnostic ability between DLB and AD when using each modality, VSRAD or CIScore, respectively [10–14]. However, to the best of our knowledge, no studies by combined use of these parameters have been reported. In the current study, we investigated which method is the best differential diagnostic ability between DLB and AD, among CIScore, the Z score of MTL and the ratio of Z score between dorsal brainstem to MTL, and the combination of these indexes. We also investigated whether the combination of the CIScore and the MRI Z score contributed to improving the differential diagnostic ability.

## Methods

### Subjects

Twenty-eight cases with DLB and 42 cases with AD were analyzed retrospectively. All cases were diagnosed by neurologists and there were no organic lesions such as cerebrovascular lesions. As for DLB, all cases were diagnosed as probable DLB based on the diagnostic criteria advocated by the 4th International Workshop [2]. The cases with AD were analyzed by the diagnostic criteria based on National Institute of Neurologic, Communicative Disorders and Stroke AD and Related Disorders Association (NINCDS-ADRDA) research group [15]. All patients were undertaken three-dimensional T1-weighted MRI using 1.5-T MRI scanners within 4 weeks before or after Tc-99m-ECD SPECT studies.

### SPECT and eZIS examination

All cases were undertaken Tc-99m-ECD SPECT, and all taken data were retrospectively analyzed statistically using easy Z score Imaging System (eZIS) (PDR radiopharma Inc. Tokyo, Japan) [16].

All SPECT scans were performed using a triple-headed gamma camera (GCA9300R; Canon Medical Systems Corp., Otawara City, Japan) and high-resolution fan-beam collimator. SPECT images were obtained with a 128 × 128 matrix and continuous repetitive data acquisition mode with 120° Rotation, acquisition time was 16 min (3 detector, 30 view/detector, 4repeat 2cycle/repeat, time/cycle 120 s). For SPECT image reconstruction, a Butterworth filter (cutoff frequency, 0.76 cycles/cm; order, 4) was used. Attenuation correction was performed using Chang’s method (*µ* = 0.15/cm) and scatter correction was performed with a triple energy window (TEW).

After the eZIS analysis, we computed the CIScore [12, 13]. This program also allows statistical parametric mapping results to be incorporated into an automated analysis of the Z score values in the volume of interest (VOI). Using eZIS program, two VOIs were set automatically on the posterior cingulate gyrus (VOI-1) and on the occipital lobe (VOI-2). The CIScore is defined as the ratio of the VOI-2 to the VOI-1, which is automatically calculated.

### MRI and VSRAD examination

In the present study, 3-dimensional T1-weighted sagittal images were taken with an Intera Achieva 1.5 Tesla (Philips, Tokyo, Japan) under the following setting: FOV 240, matrix 256 × 256, slice thickness 1.2 mm, 220 slices, TR 10 ms, TE 5 ms, flip angle 25°.

VSRAD, we assessed brain atrophy in local gray matter or white matter was a free software designed to evaluate relative local brain volume of individual patients comparing with brain MRI database for healthy individuals by means of voxel-based morphometry (VBM) [10]. Using this program, the entire brain by 3-demensional T1-weighted image taken with a 1.5-Tesla MRI device is processed with SPM8 (Statistical Parametric Mapping, 2008 Edition) to isolate grey matter based on the anatomical standardization followed by statistical analysis of grey matter density.

The results of analysis are displayed as a colored scale map on the standard brain. These Z-score maps were displayed by overlay on tomographic sections and surface rendering of the standardized brain. We registered target VOIs in the MTL and DBS as specifically atrophied areas in AD and DLB, respectively, according to previous studies [10, 17].

### Description of present method and data analyses

First, we computed the CIScore. Although the threshold value is 0.281 in the default setting, 0.26 was the best cut-off value from our receiver operating characteristic (ROC) analysis in the current study of our institute. Thus, we diagnosed as DLB in case with CIScore is 0.260 or less. Then, we calculated the differential diagnostic ability (Sensitivity: Sen, Specificity: Spe, Accuracy: ACC) of CIscore.

Second, we evaluated the Z score of the volumes of interest using VSRAD. The obtained data were analyzed using software VSRAD advance® on a PC. By the result of ROC analysis in the present study, the threshold values were determined. Namely in MTL, we diagnosed as DLB in case with the Z score is 2.06 or less. As for the ratio of Z score in DBS (gray matter) to MTL, we diagnosed as DLB in case with the value is 0.37 or more. Regarding the ratio of Z score in DBS (white matter) to MTL, we diagnosed as DLB in case with the value is 0.42 or more. Then, we calculated the differential diagnostic ability (Sen, Spe, ACC) by the Z score of MTL and by DBS.

Namely, each parameter and diagnostic criteria were as follows.

1. CIScore as a parameter of rCBF SPECT, we diagnosed as DLB in case which is 0.259 or less.
2. Z score value of MTL atrophy by MRI, we diagnosed as DLB in case which is 2.05 or less.
3. The atrophic ratio of dorsal brainstem to medial temporal gray matter (DBS) by MRI, we diagnosed as DLB in case which is 0.38 or more.
4. The atrophic ratio of dorsal brainstem to medial temporal white matter (DBS) by MRI, we diagnosed as DLB in case which is 0.42 or more.

Finally, we compared the clinical parameters and differential diagnostic ability among the single parameter and the combination of these parameters, including and omitting the elderly (over 75 years old). The clinical parameters were statistically evaluated by unpaired *t* test or chi-square test. The diagnostic accuracy was statistically evaluated using McNemar test. All statistical analyses were performed using StatFlex version 7 (Artek Osaka Japan).

## Results

The mean values of Mini-Mental State Examination (MMSE) and age were significantly higher in DLB group compared to those in AD group in the comparison of the clinical parameters. There was no statistical significance in gender and duration of diseases among subgroup (Table 1). These results were similar when the elderly cases were excluded (Table 2).

**Table 1:** Comparison of clinical parameters

	DLB (*n* = 28)	AD (*n* = 42)	*P* value
Age	77.3 ± 6.4	73.5 ± 8.0	0.04
Gender (M:F)	8:20	12:30	NS
MMSE	23.5 ± 4.9	20.2 ± 4.5	0.007
Duration of disease (years) ≦ 5/6–10/ ≧ 10	17/6/5	27/7/8	NS

**Table 2:** Comparison of clinical parameters in the cases of younger cases (< 75 years)

	DLB (*n* = 11)	AD (*n* = 20)	*P* value
Age	70.5 ± 5.5	66.9 ± 6.2	0.03
Gender (M:F)	3:8	6:14	NS
MMSE	24.3 ± 4.0	19.3 ± 5.2	0.007
Duration of disease (years) ≦5/6–10/ ≧ 10	6/3/2	15/3/2	NS

The differential diagnosis abilities of the single parameter, by CIScore was, Sen 93%, Spe 62% and ACC 74%, respectively. Similarly, Sen 93%, Spe 52% and ACC 69% by MTL, Sen 79%, Spe 60% and ACC 67% by DBS (gray matter) (Table 3). In the conditions excluding elderly cases, CIScore was, Sen 100%, Spe 65% and ACC 77%, respectively. Similarly, Sen 100%, Spe 50% and ACC 68% by MTL, Sen 100%, Spe 45% and ACC 65% by DBS (gray matter) (Table 4).

**Table 3:** Comparison of differential diagnostic ability (single parameter)

	Sensitivity	Specificity	Accuracy
CIScore	0.93	0.62	0.74
Z score of MTL	0.93	0.52	0.69
Atrophic ratio	0.79	0.60	0.67

**Table 4:** Comparison of differential diagnostic ability (single parameter, excluding the elderly)

	Sensitivity	Specificity	Accuracy
CIScore	1	0.65	0.77
Z score of MTL	1	0.5	0.68
Atrophic ratio	1	0.45	0.65

When we diagnosed by the combination of these parameters, the sensitivity was 89% in condition with (1) and (2), 79% for (1) and (3), and 75% for (2) and (3), the specificity was 81% in condition with (1) and (2), 83% for (1) and (3), and 62% for (2) and (3), the accuracy was 84% for (1) and (2), 81% for (1) and (3), and 67% for (2) and (3) (Table 5). The combination method of CIScore and Z score value of MTL atrophy showed the best accuracy. When we used this combination method and omitting the elderly, the accuracy was 94% for (1) and (2), 90% for (1) and (3), and 68% for (2) and (3) (Table 6). The accuracy of all combination of these parameters improved when we excluded the elderly (over 75 years old).

**Table 5:** Comparison of differential diagnostic ability (combination)

	Sensitivity	Specificity	Accuracy
(1) and (2)	0.89	0.81	0.84
(1) and (3)	0.79	0.83	0.81
(2) and (3)	0.75	0.62	0.67

(1) CIScore, (2) Z score of MTL, (3) atrophic ratio

**Table 6:** Comparison of differential diagnostic ability (combination, excluding the elderly)

	Sensitivity	Specificity	Accuracy
(1) and (2)	1	0.9	0.94
(1) and (3)	1	0.85	0.90
(2) and (3)	1	0.5	0.68

(1) CIScore, (2) Z score of MTL, (3) atrophic ratio

**Fig. 1:**
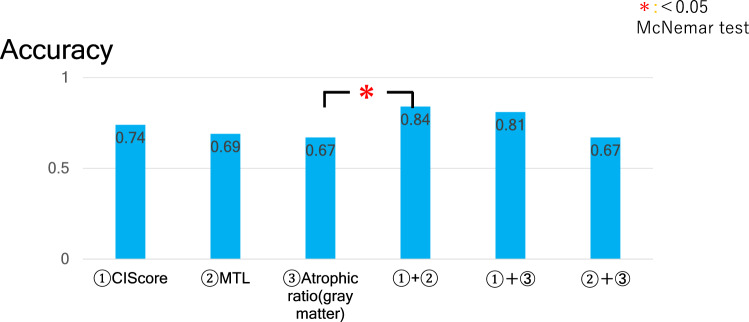
The accuracy of the combination method by CIScore or Z score was 84%, which was higher than other parameters, and significantly higher than DBS alone

**Fig. 2:**
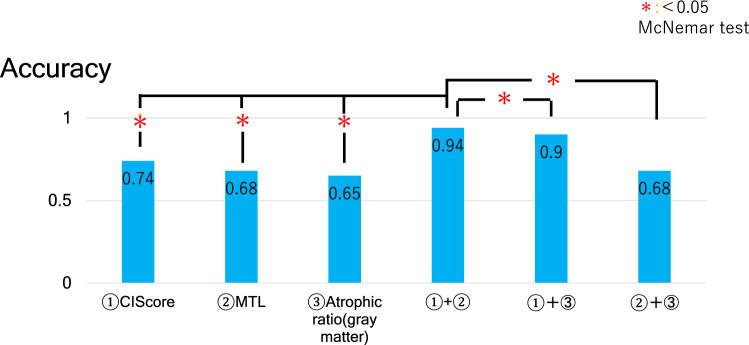
When we diagnosed confining to ages younger than 75 years old, the accuracy improved to 94%. The value was significantly higher than other parameters (single or combination)

The accuracy of the combination with CIScore and Z score value of MTL was 84%, which was higher than other parameters, and significantly higher than DBS alone (Fig. 1). When we adopted them confined to ages younger than 75 years old, the accuracy improved to 94%. This result was significantly higher than other parameters (single or combination) (Fig. 2). As for DBS, we also analyzed using white matter, the diagnostic accuracy by atrophic ratio of Z score (DBS/MTL) was lower than using gray matter. And diagnostic accuracy in each combination using DBS (white matter) was lower than those in the combination of CIScore and Z score of MTL, in both all the cases and in the group excluding elderly cases (Online Resource 1, 2).

### Cases

**Fig. 3:**
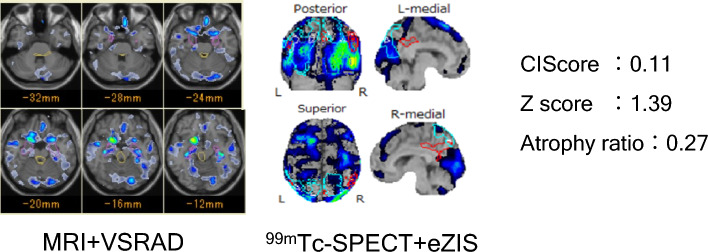
Case 1, 75 years DLB male patient with typical symptoms. The dementia scale was MMSE 13/30. CIScore was 0.11 which was true positive as DLB. The Z score of MTL using VSRAD was true positive 1.39, but DBS was false negative 0.27. The case was correctly diagnosed as DLB by the combination of CIScore and Z score of MTL

Case1 (Fig. 3). Seventy-five-year-old male diagnosed as DLBs. Insomnia was noted 8 years ago and parkinsonism with dementia from 3 years ago. The dementia level at hospitalization was MMSE 13/30. Both ^99m^Tc-ECD SPECT and 3-dimensional T1-weighted sagittal images were undertaken at hospitalized. The rCBF SPECT using eZIS analysis showed significant hypo-perfusion in the bilateral occipital lobe and the CIScore was 0.11, which was true positive diagnosing DLB. The Z score using VSRAD was true positive at 1.39, but Atrophy ratio was false negative at 0.27. This case was correctly diagnosed as DLB by the combination of CIScore and Z score.

**Fig. 4:**
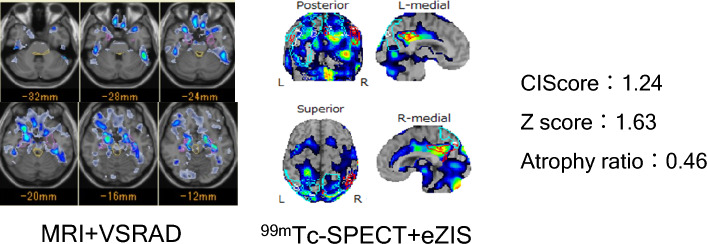
Case 2, 83 years woman patients diagnosed with AD, whose dementia scale was MMSE 22/30. The value of CIScore was 1.24 which was true negative as DLB. The Z score of MTL using VSRAD was 1.63 and DBS was 0.46. Both indexes were true negative as DLB

Case2 (Fig. 4). Eighty-three years old female with AD. Both mild dementia and pathological jealousy from 2 years ago were noted. The value of MMSE was 22/30. ^99m^Tc-ECD SPECT and 3-dimensional T1-weighted sagittal images were undertaken. The rCBF SPECT with eZIS analysis demonstrated that significant hypo-perfusion in the posterior-cingulate gyrus, and the value of CIScore was 1.24, which was a true negative. The Z score by VSRAD was 1.63, and Atrophy ratio was 0.46, both indexes showed false positive. Although we could diagnose as AD by CIScore, MRI parameters alone misdiagnosed.

**Fig. 5:**
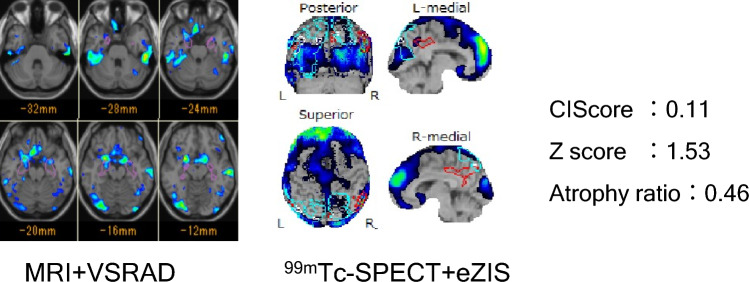
Case 3, 67 years woman patient with symptoms consistent with DLB whose dementia scale was MMSE 20/30. The value of CIScore was 0.11, true positive as DLB. Z score using VSRAD was 1.53 and the atrophy ratio was 0.46, both values were true positive as DLB

Case3 (Fig. 5). Sixty-seven years old female with DLB. She has been treated for depression for several years. Both cognitive dysfunction and visual hallucination were noted from one year ago. The dementia scale was 20/30 in MMSE and was diagnosed with DLBs. 99mTc-ECD SPECT using eZIS analysis showed that significant hypo-perfusion in the bilateral occipital lobe, and the value of CIScore was 0.11 within true positive values for DLB. As for MRI indexes using VSRAD, Z score of hippocampus was 1.53 and the atrophy ratio was 0.46, both indicated being DLB. Thus, the case was younger than 75 years old, and all parameters were true positive.

## Discussion

Both DLB and AD are common cause of dementia among the elderly populations [2]. Because of some overlaps between the two disorders in clinical characteristics and imaging features, the differential diagnosis by the combined methods would be recommended. For instances, dopamine transporter (DAT)–SPECT [18] or [123I]-metaiodobenzylguanidine (MIBG) scintigraphy [2] should be useful. AS DLB has commonly dopaminergic dysfunction and DAT SPECT shows decreased uptake of I-123 ioflupane in bilateral basal ganglia. MIBG can detect the cardiac denervation that is one of the main characteristics of DLB [19]. MRI is a valuable tool for both the diagnosis and the research of AD. Because AD is a neurodegenerative condition characterized by the gradual loss of nerve cells and changes in brain tissue, MRI plays a crucial role in non-invasively visualizing these changes [20]. Using MRI, we can assess of atrophy in specific brain areas, which is the shrinkage or loss of brain tissues. Patients with AD often exhibit progressive shrinkage particular in MTL, making it a distinctive hallmark of the disease [10, 21]. There is significantly more atrophy in DBS of DLB patients, compared to AD patients [10]. MRI can capture these changes, and it can also detect other disease, such as the infarction.

The study aimed to assess the diagnostic ability of various parameters, including the CIScore derived from rCBF SPECT and the Z score for MTL atrophy from MRI, in differentiating between DLB and AD. In addition, the study also evaluated the effect of age on the diagnostic accuracy. The accuracy of the combination with CIScore and Z score value of MTL was 84%, which was higher than other parameters, and significantly higher than DBS alone. Confined to ages younger than 75 years old, the accuracy improved to 94%. It was statistically significantly higher than other parameters (single or combination).

There are a lot of VBM investigations regarding gray matter volume changes with aging [22–24]. Most cortical regions remarkably in frontal and insular areas are known to show a linear negative association between volume and age [22–24]. In contrast, the preservation of gray matter volume in the specific structures such as amygdala, hippocampus, and thalamus are reported in many papers [22–24]. However, some degree of overlap of regional volume changes between normal aging and AD may not be avoided inevitably in the cross-sectional studies [25]. The diagnostic ability of CIScore is better in the conditions without influence of brain atrophy by aging [13].

Therefore, the diagnostic ability in both CIScore and VSRAD are affected to an extent by the brain atrophy with normal aging. Due to such backgrounds, the differential diagnostic capability by the combination with CIScore and Z score value of MTL could have been improved in the relatively younger cases such as under 75 years old in the present study.

As for the diagnostic accuracy of DBS, it was around 65% same as previous studies [26]. They also showed similar results as in Z score of MTL. However, the combined use of DBS and CIScore improved accuracy in cases under 75 years old. The result also indicated that combination of MRI and SPECT is useful in the differential diagnosis between DLB and AD younger than 75 years old.

The limitation of the present study is that the number of analyzed cases is small and the study in one institute. Future studies should be prospectively done including clinically equivocal cases by following up continuously in multi centers. In addition, Amyloid PET has come to be used for clinical settings in these days in Japan. Even in the cases of clinical diagnosis were AD, some cases were reported to be negative for Amyloid PET. Therefore, clinical diagnoses might not always be done accurately [27]. The future SPECT study regarding AD might be preferable analyzed in cases confirmed by Amyloid PET.

## Conclusion

The combination of CIScore and Z score of MTL was suggested to be useful in the differential diagnosis between DLB and AD, especially, younger than 75 years old.

## Supplementary Information

Below is the link to the electronic supplementary material.

Supplementary file1 Online Resource 1. The accuracy of the combination method by CIScore or Z score was 84%, which was higher than other parameters, and significantly higher than that (57%) of atrophic ratio using DBS (white matter). Online Resource 2. When we diagnosed confining to ages younger than 75 years old, the accuracy improved to 94%. The value was significantly higher than other parameters (single or combination) (PPTX 69 KB)

## Data Availability

The data that support the findings of this study are not openlyavailable due to reasons of sensitivity and are available from thecorresponding author upon reasonable request. Data are located incontrolled access data storage at Fukuoka University Hospital.
