# Supplementary material for: The usefulness of combined analysis using CIScore and VSRAD parameters for differentiating between dementia with Lewy body and Alzheimer’s disease
Source: Jpn J Radiol. 2024 Jun 10;42(10):1206–12. doi: 10.1007/s11604-024-01604-5 (PMC11442568; doi:10.1007/s11604-024-01604-5)
Supplement: Supplementary file 1 — Supplementary file1 Online Resource 1. The accuracy of the combination method by CIScore or Z score was 84%, which was higher than other parameters, and significantly higher than that (57%) of atrophic ratio using DBS (white matter). Online Resource 2. When we diagnosed confining to ages younger than 75 years old, the accuracy improved to 94%. The value was significantly higher than other parameters (single or combination) (PPTX 69 KB) [file 11604_2024_1604_MOESM1_ESM.pptx]

## Slide 1
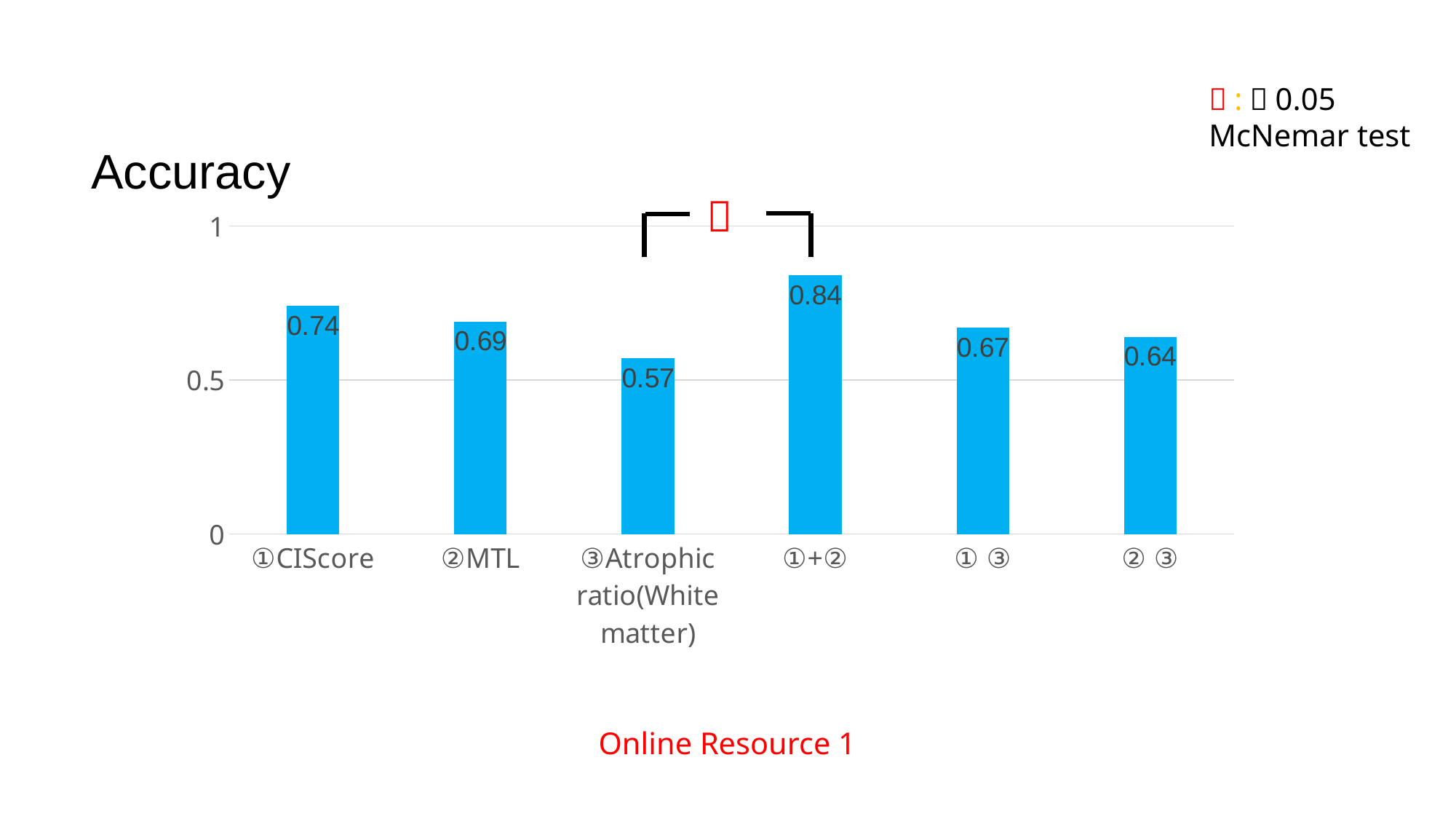

＊:＜0.05
McNemar test
Accuracy
＊
### Chart
| Category | 感度 |
|---|---|
| ①CIScore | 0.74 |
| ②MTL | 0.69 |
| ③Atrophic ratio(White matter) | 0.57 |
| ①+② | 0.84 |
| ①＋③ | 0.67 |
| ②＋③ | 0.64 |Online Resource 1

## Slide 2
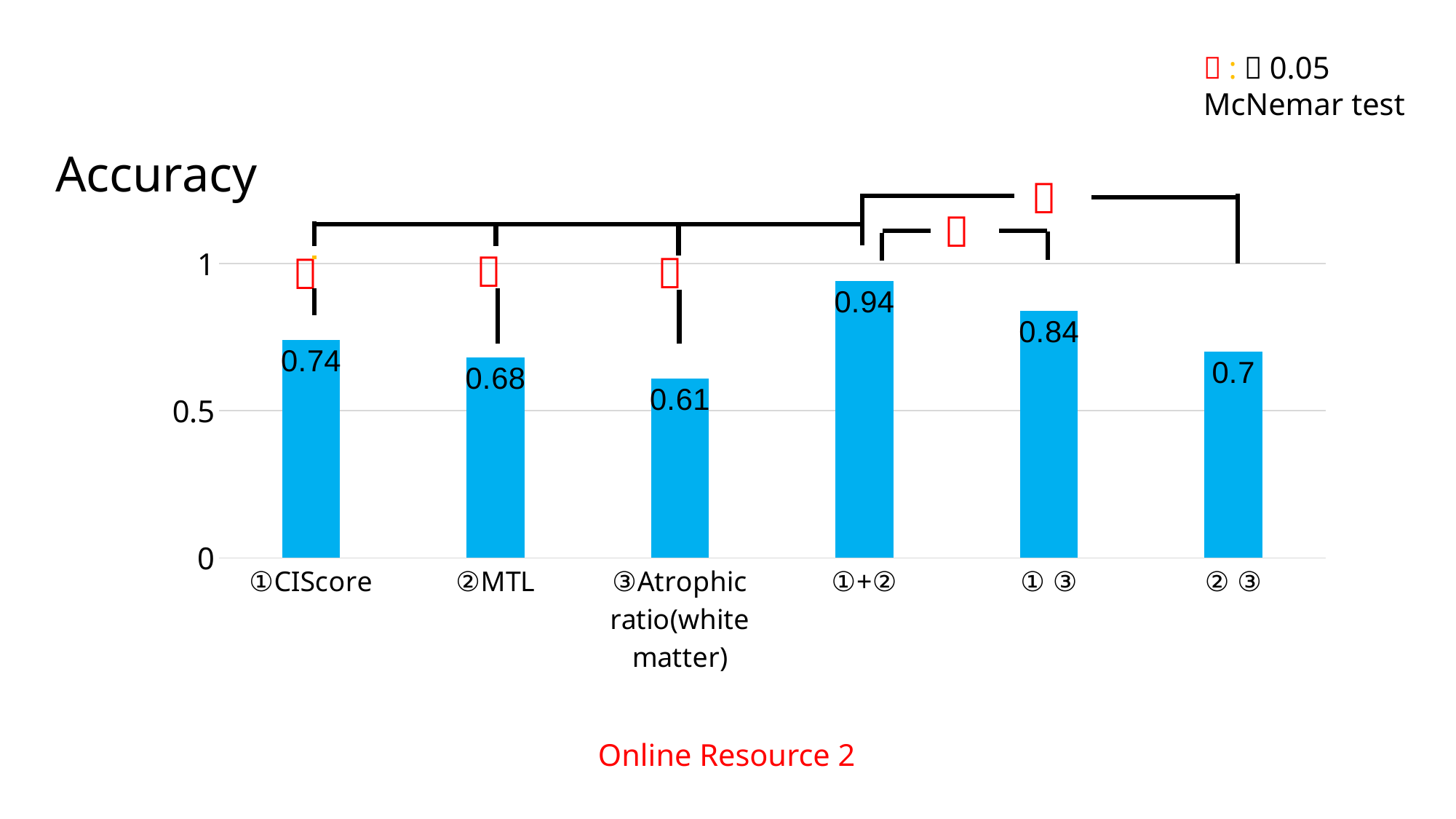

＊:＜0.05
McNemar test
Accuracy
＊
＊
### Chart
| Category | 感度 |
|---|---|
| ①CIScore | 0.74 |
| ②MTL | 0.68 |
| ③Atrophic ratio(white matter) | 0.61 |
| ①+② | 0.94 |
| ①＋③ | 0.84 |
| ②＋③ | 0.7 |＊
＊
＊
Online Resource 2
